# Human umbilical cord blood-derived mesenchymal stem cell transplantation for the treatment of spinal cord injury

**DOI:** 10.3892/etm.2014.1608

**Published:** 2014-03-06

**Authors:** BINGZHOU CUI, EN LI, BO YANG, BO WANG

**Affiliations:** 1 Department of Neurosurgery, Zhengzhou People’s Hospital, Zhengzhou, Henan 450003, P.R. China; 2 Department of Neurosurgery, The First Affiliated Hospital, Zhengzhou University, Zhengzhou, Henan 450003, P.R. China

**Keywords:** human umbilical cord blood, mesenchymal stem cells, spinal cord injury, transplantation, rat

## Abstract

The aim of the present study was to investigate the effects of human umbilical cord blood-derived mesenchymal stem cell (HUCB-MSC) transplantation on the functional restoration of spinal cord injury (SCI). A total of 46 adult Wistar rats were randomly divided into three groups: Injury (n=15), control (n=15) and transplantation (n=16). A SCI model was established using the modified Allen’s method (vulnerating energy, 25 g/cm). The rats in the control and transplantation groups were injected at the site of the injury with physiological saline and HUCB-MSC suspension, respectively. At week one, two and four following treatment, the behavior of the rats was evaluated using the Basso, Beattie, Bresnahan locomotor rating scale. In addition, immunohistochemistry (IHC) was performed on samples from the rats that had been sacrificed four weeks subsequent to the treatment. Recovery of the spinal cord nerve function was identified to be significantly different at week two and four following treatment (P<0.05), and IHC identified that at week four following treatment novel nerve cells were being produced. Thus, transplantation of HUCB-MSCs promoted the recovery of the damaged function of spinal cord nerves in rats with SCI.

## Introduction

Spinal cord injury (SCI) is a common type of severe trauma. Maximum recovery of spinal cord function has become increasingly studied for the treatment of SCI. It was previously hypothesized that following injury, the central nerve is not able to regenerate; however, recent studies have identified that by altering the local environment following SCI, the injured nerve axons are able to regenerate and partial functioning of the spinal cord can be restored (1,2). The following methods alter the local environment of the spinal cord following injury and aid with the regeneration of injured nerve axons (3): Transplantation of fetal spinal cord tissue, peripheral nerves, neural stem cells, Schwann cells, macrophages, olfactory ensheathing cells and umbilical cord blood stem cells (4–13), transplantation of fibroblasts with a neurotrophic factor injection or secretion (14,15) and removal of myelin-related nerve growth inhibitory factors.

Previous studies have identified that marrow mesenchymal stem cells (MSCs) can be differentiated into neural cells *in vitro* and *in vivo* via induction. This provides a novel method for the treatment of SCI and has been shown to be effective in certain clinical applications (16–18). Previously, MSCs were found to exist in the cord blood and be induced to differentiate into bone, fat or neuron-like cells in certain conditions or when cultured *in vitro*; indicating that cord blood-derived MSCs have the potential to differentiate into a variety of cells, including nerve cells (19–22). Clinical trials of stem cell transplantation for the treatment of a number of nervous system disorders have been conducted for several years, achieving positive results (23) and providing confidence in current stem cell research and its clinical applications for the treatment of SCI. Several previous studies have indicated that stem cell transplantation effectively promotes the repair of the spinal cord in animals, thus, reducing SCI (24–26). Although numerous studies have been performed on cord blood-derived MSCs (6,27,28), few have focused on the applications of MSCs. In the present study, human umbilical cord blood (HUCB)-MSC transplantation was used for the treatment of a rat SCI model. The behavior and histological changes exhibited by the rats were investigated to evaluate the therapeutic effect of HUCB-MSC transplantation. The aim of the current study was to identify a type of cell that is suitable for the treatment of SCI and to provide further experimental evidence for its clinical application.

## Materials and methods

### HUCB

HUCB was obtained from the Departments of Gynecology and Obstetrics at the First and Third Affiliated Hospitals of Zhengzhou University and Zhengzhou People’s Hospital (Zhengzhou, China). HUCB was obtained from healthy full-term cesarean or full-term eutocia puerpera patients who tested negative for the hepatitis B virus. The present study was conducted in accordance with the Declaration of Helsinki and with approval from the Ethics Committee of Zhengzhou University. Written informed consent was obtained from all the participants.

### Animals

In total, 46 healthy adult female Wistar rats (weight, 250–280 g) were obtained from the Experimental Animal Center of Henan (Zhengzhou, China). The rats were housed in a specific pathogen-free room at a constant temperature of 25°C and humidity of 45%. The present study was conducted in strict accordance with the recommendations in the Guide for the Care and Use of Laboratory Animals of the National Institutes of Health. The animal use protocol was reviewed and approved by the Institutional Animal Care and Use Committee of The First Affiliated Hospital of Zhengzhou University.

### Model establishment and grouping

The SCI model was established in accordance with the modified Allen’s method. The rats were anesthetized with 400 mg/kg chloral hydrate via intraperitoneal injection. A laminectomy was subsequently performed on the entire spinous process, the vertebral plate of T9 and part of the vertebral plate of T8 and T10, to expose the dorsal (posterior) surface of the spinal cord. The exposed spinal cord at the T9 level was vulnerated with a 10 g weight dropped from a height of 2.5 cm (vulnerating energy, 25 g/cm). Following injury, the rats were randomly divided into three groups. The injury group (n=15) received no treatment following injury, the control group (n=15) were treated with physiological saline and the transplantation group (n=16) were treated with the HUCB-MSC suspension.

### Cell isolation, culture and identification

HUCB 50–80-ml samples were collected in a blood collection bag containing composite citrate phosphate dextrose adenine-1 anticoagulant under sterile conditions and stored at 4°C. The samples were isolated within 12 h of collection using Ficoll-Hypaque, according to the manufacturer’s instructions. HUCB was diluted with physiological saline (1:1), placed in the Ficoll-Hypaque and centrifuged at 626 × g for 20 min (the centrifuge was provided by Hunan Xingke Medical Scientific Instrument Co., Ltd., Changsha, China). Following centrifugation, the cord blood mononuclear cells, which contained MSCs, were collected. The HUCB was removed, placed in a second tube and washed three times in physiological saline. The cells were stained with trypan blue and the viable cells were counted under an optical microscope (Nikon Corp., Tokyo, Japan). HUCB mononuclear cells were diluted to a cell density of 1.0×10^6^/ml with a specialized MSC medium (Stemcell Technologic Inc., Vancouver, Canada) and placed in an incubator with a 5% CO_2_ atmosphere and saturated humidity of 37°C. After six days, the culture media was removed and changed, then subsequently changed every three to four days. When cell growth was exponential, the cells were passaged at a 2:1 ratio for the two initial passages and then at a 1:1 ratio for the third passage.

### Preparation and implantation

The third passage of the HUCB-MSCs cultured *in vitro* was collected and diluted to a density of 1.0×10^7^/ml. A 5-μl cell suspension was implanted into the wounded site of the rats with SCI. The control group underwent the same procedure using physiological saline.

### Behavior and histological changes

At week one, two and four following transplantation, an inclined plane test was conducted and Basso, Beattie, Bresnahan (BBB) locomotor rating scale (29,30) values were obtained for the rats in the control and transplantation groups. Samples collected from the rats at week one and four were stained with hematoxylin and eosin (HE) or by immunohistochemistry (IHC), to examine the histological changes (the related kits and reagents were provided by Beijing Zhongshan Biotechnology Co., Ltd., Beijing, China).

### Statistical analysis

Statistical analysis was performed using SPSS software 10.0 (SPSS, Inc., Chicago, IL, USA). Data are expressed as the mean ± SD. Differences among the groups and different time periods were compared using the t-test and P<0.05 was considered to indicate a statistically significant difference.

## Results

### Isolation of HUCB-MSCs and culture

The mononuclear cells that were isolated from the HUCB consisted of two types of cell; a small number of spindle-like cells and a large number of osteoclast-like cells. Osteoclast-like cells were large, round or oval-shaped and possessed multiple nuclei. The majority of the spindle-like cells were HUCB-MSCs, which were successfully isolated from 18 of the 32 samples of HUCB, however, only four were amplified and cultured *in vitro*.

In the early stages of culture, the HUCB-MSCs were round. However, when the quantity of adherent cells increased, the cell body gradually became spindle-like. The HUCB-MSCs were mononuclear and predominantly distributed in a diffuse pattern, with a small number growing in colonies. When cultured *in vitro,* a number of the MSCs developed into heterogeneous adherent cells. The cells varied in shape, exhibiting small and round structures or irregular shapes; a number of the cells were shaped like a poached egg or a star and certain cells were large with multiple nuclei. Approximately three weeks after culturing, with the rapid proliferation of the cells, the HUCB-MSCs appeared to be relatively uniform, exhibiting long spindle-like structures and colony distribution. Once the cells had grown to 80–90% confluence, they were harvested and inoculated in passage culture flasks. After 15 days, the cells were subcultured and amplified to the third passage; the HUCB-MSCs were implanted in the rats with SCI, according to the methods described previously by Wang *et al* (31).

### Animal behavior

Normal rats were graded on a 21-point scale, according to the BBB ratings, prior to surgery (30,31). Following surgery and transplantation, the rats in the three groups were graded at various time points. At day one after the induction of SCI, the rats scored zero points. After one week, the scores improved, although no significant differences were identified among the three groups (P>0.05). At week two following treatment, the BBB ratings of the rats in the transplantation group were greater than that of the injury and control groups (P<0.05). In addition, at week four following treatment, the BBB ratings of the rats in the transplantation group exhibited improved recovery when compared with those in the other groups (P<0.05). The rats were able to stand on their hind limbs and exhibited concordant movements with their fore and hind limbs (Table I).

### Neuron-specific enolase (NSE) and glial fibrillary acidic protein (GFAP) expression

At week one, two and four following treatment, the HE and IHC staining were performed on the spinal cord tissue of rats. HE staining results identified that the SCI had resulted in a marked inflammatory reaction at week one; there was a large quantity of inflammatory cell infiltration. At week four following treatment, there was a degree of inflammatory cell infiltration and a marginal difference was observed in the HE sections between the control and transplantation groups.

**Figure 1:**
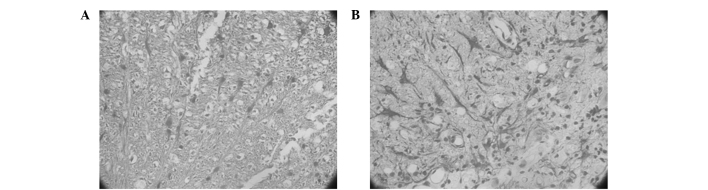
(A) NSE and (B) GFAP expression in the spinal cord of rats in the transplantation group, as detected by IHC staining at week 4 following treatment (A, magnification, ×100; B, magnification, ×400. NSE, neuron-specific enolase; GFAP, glial fibrillary acidic protein; IHC, immunohistochemistry.

The expression of NSE and GFAP in the spinal cord tissue was detected by IHC. There were virtually no NSE^+^ or GFAP^+^ cells identified in the injury and control groups at week four following the treatment; however, there was a small number of NSE^+^ cells and a large number of GFAP^+^ cells observed in the transplantation group. The NSE^+^ cells appeared in a streak or group-like manner and there were small protrusions around the NSE^+^ cells. The GFAP^+^ cells were stained a dark color and exhibited morphological diversity. The processes of the GFAP^+^ cells increased in length and a number of the cells were fibrous and dendritic cell-like, becoming interlaced into a network within the spinal cord (Fig. 1).

## Discussion

In the present study, HUCB-MSCs were effectively isolated and amplified *in vitro,* and growth was observed in the specialized MSC medium. Redundant MSCs were obtained through passaging, which may provide the experimental foundation for future investigations and clinical applications.

The results of the present study demonstrated that HUCB-MSC transplantation for the treatment of rat SCI significantly improved the neurological function of the damaged spinal cord at week two following treatment. In addition, at week four after treatment, further improvement was observed, which was consistent with the results of previous studies regarding the treatment of SCI by transplantation of MSCs (32,33). Thus, the results indicate that the damaged spinal cord may recover and regenerate following the implantation of HUCB-MSCs. However, the underlying mechanisms behind how the transplanted MSCs survive, concentrate and migrate to the damaged spinal cord, remain unclear. The present study indicated that at week two following HUCB-MSC transplantation into the damaged spinal cord, the MSCs adapted to the microenvironment of the spinal cord and spontaneously secreted or induced other cells to produce nerve repair factors. This promoted local nerve repair and stimulated the surviving nerve axons to extend their lateral branches towards the damaged axons, resulting in improved function of the spinal cord nerves (34–36). In addition, the mechanism may be associated with the integration of the transplanted cells into the neural circuits (37,38). The IHC results at week four identified that a number of the implanted MSCs transformed into nerve cells and were able to survive for long periods of time. The MSCs adapted to the environment of the body and continued to promote the recovery of the injured spinal cord. The HE results at week one and four revealed that immune rejection of the MSC transplantation was not significantly different from the group with non-implanted cells. Therefore, HUCB-MSC transplantation for the treatment of rats with SCI is considered to be a safe method.

In conclusion, the present study identified that MSCs can be isolated from HUCB, cultured and passaged *in vitro*. Following transplantation of the passaged MSCs into rats with SCI, the isolated MSCs were able to survive in the bodies of the rats without experiencing immune rejection. The implanted MSCs were able to differentiate into nerve cells, which was involved in the recovery of the damaged spinal cord. Thus, the BBB locomotor rating scale scores were improved and the recovery of motor function following SCI was promoted. Therefore, the results provide a theoretical and experimental basis for HUCB-MSC transplantation for the treatment of SCI. Following further investigation, it may be possible to apply HUCB-MSC transplantation to the clinical treatment of SCI. Compared with other stem cells, HUCB-MSCs exhibit certain advantages, including favorable primitiveness, strong amplification ability, simple acquisition and weak *in vivo* rejection, without any damage to the donor. Therefore, HUCB-MSCs may provide a novel cell source and method for the treatment of SCI.

## Figures and Tables

**Table I:** BBB locomotor ratings of the rats in the three groups.

	BBB locomotor ratings				
	
Group	Day 1	Week 1	Week 2	Week 3	Week 4
A	0	6.6±0.7	9.6±1.2	10.1±1.3	11.0±1.5
B	0	6.4±0.5	9.4±1.3	9.8±1.6	10.9±1.8
C	0	7.3±0.6	12.8±1.6^a^	13.5±2.1^a^	14.2±2.3^a^

A, injury group; B, control group; C, transplantation group.

a P<0.05, group C compared with both groups A and B.

Data are expressed as the mean ± SD. BBB, Basso, Beattie, Bresnahan.
